# Beneficial Effect of Sodium Nitrite on EEG Ischaemic Markers in Patients with Subarachnoid Haemorrhage

**DOI:** 10.1007/s12975-021-00939-9

**Published:** 2021-09-07

**Authors:** Alexander Luettich, Edit Franko, Desiree B. Spronk, Catherine Lamb, Rufus Corkill, Jash Patel, Martyn Ezra, Kyle T. S. Pattinson

**Affiliations:** 1 Nuffield Department of Clinical Neurosciences, Oxford University, Oxford OX3 9DU, UK; 2 Neuro Intensive Care Unit, John Radcliffe Hospital, Oxford University Hospitals NHS Foundation Trust, Oxford, UK; 3 Department of Neuroradiology, John Radcliffe Hospital, Oxford University Hospitals NHS Foundation Trust, Oxford, UK; 4 Department of Neurosurgery, John Radcliffe Hospital, Oxford University Hospitals NHS Foundation Trust, Oxford, UK

**Keywords:** Subarachnoid haemorrhage, Nitrite, Nitric oxide, EEG, Alpha-delta power ratio

## Abstract

Subarachnoid haemorrhage (SAH) is associated with long-term disability, serious reduction in quality of life and significant mortality. Early brain injury (EBI) refers to the pathological changes in cerebral metabolism and blood flow that happen in the first few days after ictus and may lead on to delayed cerebral ischaemia (DCI). A disruption of the nitric oxide (NO) pathway is hypothesised as a key mechanism underlying EBI. A decrease in the alpha-delta power ratio (ADR) of the electroencephalogram has been related to cerebral ischaemia. In an experimental medicine study, we tested the hypothesis that intravenous sodium nitrite, an NO donor, would lead to increases in ADR. We studied 33 patients with acute aneurysmal SAH in the EBI phase. Participants were randomised to either sodium nitrite or saline infusion for 1 h. EEG measurements were taken before the start of and during the infusion. Twenty-eight patients did not develop DCI and five patients developed DCI. In the patients who did not develop DCI, we found an increase in ADR during sodium nitrite versus saline infusion. In the five patients who developed DCI, we did not observe a consistent pattern of ADR changes. We suggest that ADR power changes in response to nitrite infusion reflect a NO-mediated reduction in cerebral ischaemia and increase in perfusion, adding further evidence to the role of the NO pathway in EBI after SAH. Our findings provide the basis for future clinical trials employing NO donors after SAH.

## Introduction

Aneurysmal subarachnoid haemorrhage (SAH) is a sub-type of stroke related to the accumulation of blood in the subarachnoid space, caused by a rupture of an intracranial aneurysm [1]. Patients often remain highly disabled long term, showing cognitive and emotional impairments, failure to return to work and a reduction in quality of life [2]. Accordingly, aneurysmal SAH accounts for a disproportionally high share of costs of all cerebrovascular diseases in the UK and is associated with a serious decrease in life expectancy [3].

Pathophysiological changes after the ictus occur in two stages. A phase of early brain injury (EBI) [4, 5] can be followed by a stage of delayed neurological deterioration often including delayed cerebral ischaemia (DCI) [6, 7]. EBI describes the acute effects of intracranial haemorrhage and transient global cerebral ischaemia within 72 h of the bleed and includes (1) changes in physiology related to increased intracranial pressure, decreased cerebral blood flow and impaired cerebral autoregulation; (2) hydrocephalus, mechanical stress on the subarachnoid space contributing to vessel constriction; and (3) ionic, molecular and cellular changes promoting oxidative stress, inflammation, platelet aggregation, endothelial dysfunction, vessel constriction and cytotoxicity [5]. DCI mostly occurs within 4–10 days after the initial bleed [8] in approximately 30% of SAH patients and is—in combination with infarction from DCI—among the most unfavourable factors for outcome after SAH [6]. As angiographic vasospasm has been questioned as the most important factor leading to the development of DCI, more recent research increasingly considers a complex and multifactorial aetiology of DCI plausibly related to EBI disease mechanisms originating before DCI will usually occur [4–7].

A disruption to the nitric oxide (NO) pathway may play a pivotal role in the pathogenesis of EBI and DCI [9]. NO is an endogenous vasodilator and signalling molecule, is part of the neurovascular unit and is involved in regulating cerebral blood flow [10, 11]. Following SAH, there are changes in cerebral NO levels, yet the significance of these changes for outcome remains inconclusive [12–15]. Endogenous nitrite represents an important storage pool for NO, where nitrite can be reduced to NO under hypoxic and acidic conditions [16, 17]. Animal research points to the potential of NO to act toward a decrease in ischaemic brain injury after cerebral haemorrhage [11]. NO donor administration in rats suggests that NO can increase cerebral blood flow and prevent early ischaemic damage after experimental SAH [18, 19] and can have a neuroprotective effect in ischaemia/reperfusion models [20, 21]. Sodium nitrite infusion in monkeys was shown to prevent cerebral arteriographic vasospasm after SAH [22]. Importantly, sodium nitrite infusion can safely modulate systemic nitrite levels in humans [23]. This raises the question if sodium nitrite infusion can modulate or even protect brain function after human cerebral insult. An uncontrolled electroencephalography (EEG) pilot study by Garry et al. [24] suggested that the NO pathway after human SAH is potentially manipulable by sodium nitrite infusion.

EEG provides immediate information on neuronal functioning and connects with cerebral perfusion and brain metabolism [25–28]. It is also a sensitive tool for detecting ischaemic changes after SAH. Specifically, a decrease in the alpha-delta power ratio (ADR) has been related to cerebral ischaemia [29–32]. We conducted an experimental medicine study probing the role of the NO pathway during the EBI period after human SAH. We tested the hypothesis that sodium nitrite infusion would lead to an increase in ADR power.

## Methods

### Patients

The study protocol was approved by the South Central–Oxford C NHS Health Research Authority Ethics Committee (reference number: 12/SC/0366). Patients were identified by members of their clinical team and recruited from the acute SAH admissions to the John Radcliffe Hospital in Oxford. In order to obtain a maximally representative sample of patients, inclusion and exclusion criteria were intentionally kept to a minimum (inclusion criteria: Age range 18–80 years, aneurysm secured by intravascular coiling, all World Federation of Neurological Surgeons Subarachnoid Haemorrhage Grading (WFNS) scores, study conducted within a maximum of 5 days after the ictus; exclusion criteria: Contraindications to sodium nitrite, pre-existing aneurysmal clip). SAH was diagnosed via lumbar puncture or CT. Informed consent was given either by the patient or a personal consultee who acted in the best interest of the patient.

### Sample Size

Sample size calculation was pre-registered (https://osf.io/ywa9r/) and based on an uncontrolled pilot study by Garry et al. [24] who investigated EEG responses to nitrite following SAH. We focused the study on patients who did not subsequently develop DCI, as a robust response to nitrite was observed in this group, and this patient group was expected to represent approximately 70% of cases. Garry et al. showed large increases in ADR power after sodium nitrite infusion in seven patients who did not develop DCI (baseline ADR: *Mean* 0.037, *SD* 0.023; infusion ADR: *Mean* 0.063, *SD* 0.048). Based on these data, a calculation of the required sample size to replicate this effect was conducted (mean of paired differences: 0.027, *SD* of paired differences: 0.027). Assuming 80% power and a level of significance of 5% (two-sided), there should be an effect with 11 patients who did not develop DCI. As DCI is expected to occur in 30% of cases [7] and there was a 1:1 allocation ratio of sodium nitrite and saline infusion, recruitment of 32 patients should suffice to replicate the effect.

The sample size calculation was based on within patient changes in ADR. The present investigation moved beyond investigating within patient ADR changes, comparing ADR changes over time between groups of sodium nitrite and saline infusion.

### Randomisation and Infusion

The study was a double-blind (patients and experimenters) and placebo-controlled experimental medicine study. Patients were allocated 1:1 to either the experimental or control group by random sealed envelope. Randomisation codes were used by an independent doctor or nurse who accordingly prepared either a sodium nitrite (10mcg/kg/min, experimental condition) or saline (0.9% sodium chloride, control condition) infusion. Dosing schemes and timing of infusion were informed by a pilot study in subarachnoid haemorrhage patients and a study in healthy participants [24, 33]. Infusions were administered during EEG at an equivalent rate (0.12 ml/kg/h) by a suitably trained clinician via a peripherally sited intravenous cannula (Becton Dickinson Venflon, 20 gauge) in the patient’s arm using a calibrated infusion pump (CareFusion Alaris PK). EEG data was analysed by a researcher who was unblinded to patient randomisation codes in two stages. In a first step, allocation of patients to two groups was unblinded after a statistical analysis plan was published (https://osf.io/ywa9r/). At this stage, group identity was defined by a place holder. In a second step, allocation of the two groups to either sodium nitrite or saline infusion was unblinded after final analyses.

### Procedures

Patients received standard medical care independently of their inclusion in the study. WFNS score on admission, Fisher scale and Glasgow Coma Scale (GCS) on study day were collected (for a more comprehensive description of assessments, see supplemental methods, assessments). We conducted the study as soon as possible after successful aneurysm treatment with endovascular coiling within 5 days after the ictus at John Radcliffe Hospital in Oxford. Medical records were assessed by an independent consultant neurointensivist, according to the definitions of Vergouwen et al. [8] to determine if patients had developed DCI. Patients did not show evidence of DCI at or before the time of the study.

On the study day, the EEG was recorded for 20 min before and 60 min during infusion of sodium nitrite or saline. Drugs used for sedation were recorded as sedation is known to influence the EEG [34–38]. The EEG (for a detailed description, see supplemental methods, EEG) was acquired from electrodes Fp(1,2), F(3,4,z), T(3,4), Cz, P(3,4,z) and Oz/O1. The EEG was referenced to the average left and right mastoid recording, pre-processed, cleaned, decomposed and averaged within the alpha (8–12 Hz) and delta (1–3.5 Hz) frequency power range, and the alpha-delta power ratio (ADR) was calculated. The alpha, delta and ADR signals were then averaged within a 20-min baseline time window before and within a 60-min or three successive 20-min infusion time windows after the onset of the infusion.

As the magnitude of the EEG signal depends heavily on several factors (conductivity between electrodes and the scalp, neural and non-neural tissue) that can vary substantially between patients and experimenters, the signal was scaled according to the following equation:

$$percentage\;signal\;change=100\;x\frac{\left(power\;during\;infusion)-(power\;during\;baseline\right)}{power\;during\;baseline}$$

### Statistical Analysis

We concentrated our analysis on patients who did not develop DCI (see the “Sample Size” section). As patients who develop DCI may react differently to nitrite infusion [24] and their number was very low in our study, these patients could not reasonably be included as a separate group in a larger model together with patients who did not develop DCI. Accordingly, we initially evaluated the effect of sodium nitrite infusion across both patient groups and further investigated effects statistically for patients who did not develop DCI and qualitatively for individuals who developed DCI.

The Stata 14 software package (StataCorp 2015) was used for statistical analyses. In a first step, we conducted simple linear regression investigating the effect of nitrite infusion on the EEG controlling for potentially confounding variables: The depedent variable was percentage EEG change, independent variables included infusion type (nitrite/saline), baseline EEG and the presence of sedation (yes/no). In order to study the effect of sodium nitrite on the EEG over time, we employed a multilevel linear regression model. The dependent variable was percentage EEG change. Independent variables comparised infusion type, time window (0–20 min, 20–40 min, 40–60 min), the interaction of these two variables, baseline EEG and sedation. Patients were included as a random effect. For simple and multilevel regression, sets of matched models were used to investigate the effect of sodium nitrite on ADR, alpha and delta frequency power.

## Results

### Patients

**Fig. 1:**
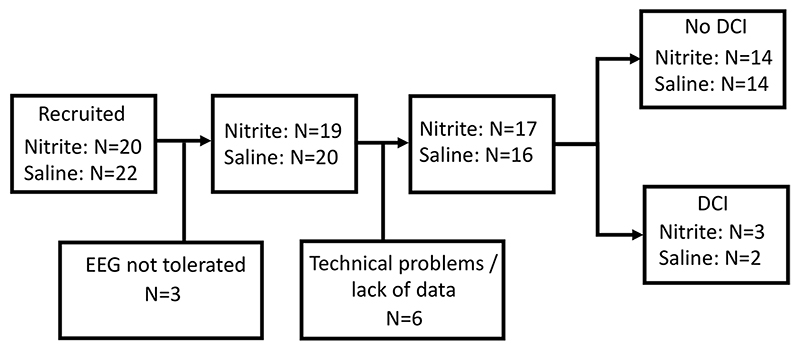
Flow chart of recruited patients

Forty-two patients were recruited. No patients were excluded for any characteristic. However, for several patients, no data or only an insufficient amount of clean data could be obtained: Three patients did not tolerate EEG recording, and there were technical problems or there were no data after data cleaning with six patients, leaving 33 patients (mean years of age 50.64 [29–69], 21 women, median WFNS score 1 [1–2]) for the analysis. Five of these patients developed DCI (mean years of age 55.2 [43–66], 3 women, median WFNS score 2 [1–2]), 28 did not develop DCI (mean years of age 49.82 [29–69], 18 women, median WFNS score 1 [1–2]). A flow chart of recruited patients including patient allocation to either sodium nitrite or saline infusion is depicted in Fig. 1. No drug-related adverse events were identified.

As access to the back of the head was challenging for practical and medical reasons, availability of electrodes was biased toward frontal and medial electrodes but was comparable for the two infusion groups (supplemental table I). Summary patient characteristics for patients who did not develop DCI are depicted in Table 1. Patients who received the nitrite infusion were largely matched in baseline characteristics of age, sex, time from bleed and Fisher scale to patients who received the saline infusion. None of the patients of the saline arm was sedated. Three patients of the nitrite arm were sedated (using fentanyl and propofol or midazolam), and this was explicitly respected in our statistical models. These patients also featured a lower Glasgow Coma Scale (GCS) on study day (E1VTM1) and accounted for the two datasets with higher WFNS scores (4 and 5) on admission. EEG power at baseline was largely comparable between patient groups but was explicitly accounted for in the statistical models.

Individual characteristics for patients who developed DCI were generally variable and are depicted in Table 2. Patient 2 was sedated (using fentanyl) and showed comparably high delta power at baseline.

### Statistical EEG Analyses Across All Patients

Results for simple linear regression (see supplemental figures I-III for model residuals) evaluating the effect of nitrite across all patients showed relative percentage change increases in ADR and alpha power with sodium nitrite versus saline infusion (difference %-change ADR: 37.75, *SE* = 15.10, *p* = 0.018, 95% CI = [6.86, 68.64]; difference %-change alpha: 28.60, *SE* = 11.14, *p* = 0.016, 95% CI = [5.82, 51.38]). No effect of sodium nitrite infusion on percentage delta power change could be observed, and no effect of baseline was found for any EEG measure.

### Statistical EEG Analyses in Patients Who Did Not Develop DCI

Further statistical analyses concentrated on patients who did not develop DCI. For simple linear regression (see supplemental figures IV-VI for model residuals), we observed relative percentage change increases in ADR and alpha power with nitrite versus saline infusion (difference %-change ADR: 51.57, *SE* = 17.68, *p* = 0.008, 95% CI = [15.08, 88.06]; difference %-change alpha: 37.46, *SE* = 12.37, *p* = 0.006, 95% CI = [11.92, 63.00]). There was no effect of nitrite infusion on percentage delta power change. Sedation resulted in a decrease in alpha power change (difference %-change alpha: - 46.94, *SE* = 20.64, *p* = 0.032, 95% CI = [- 89.55, - 4.33]), but had no effect on the ADR or delta. No effects of baseline were observed for any EEG measure.

The effect of nitrite on ADR, alpha and delta power was also investigated over time using multilevel regression (see supplemental table II for number of data sets per time window, see supplemental figures VII-XII for model residuals including model residuals for control analyses and supplemental tables III-V for control analyses).

**Fig. 2:**
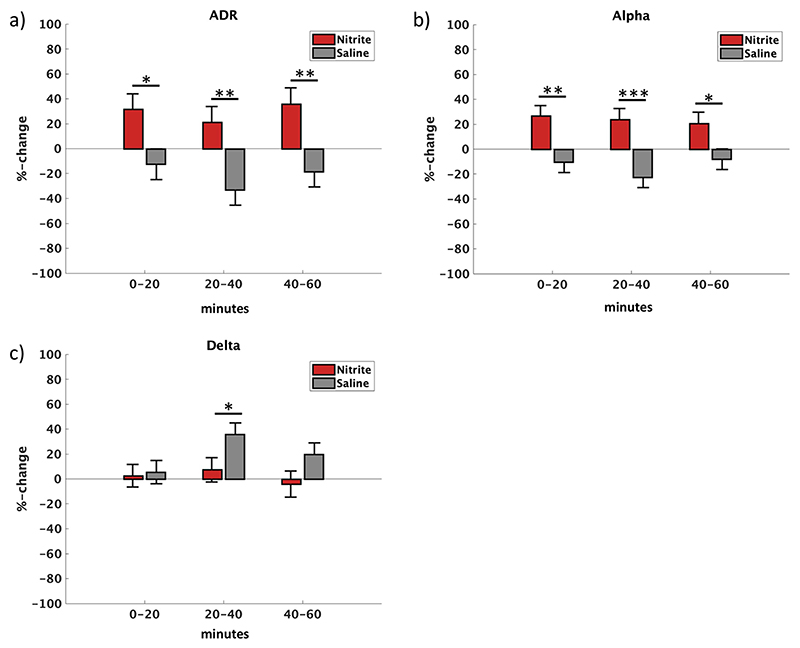
Statistical EEG analyses in patients who did not develop DCI. Predictive margins for percentage change ADR (**a**), alpha (**b**) and delta (**c**) power during nitrite (red) and saline (grey) infusion for individual time windows. Marginal effects between groups are marked with stars: ***: *p* < 0.001, **: *p* < 0.01, *: *p* < 0.05. Error bars denote standard error

There was a difference in ADR power change between infusion groups for all three time windows (Fig. 2a), where there was more ADR power change in the sodium nitrite group (marginal effect %-change, time window 0–20 min: 44.27, *SE* = 17.22, *p* = 0.013, 95% CI = [9.34, 79.20]; marginal effect %-change, time window 20–40 min: 54.37, *SE* = 18.05, *p* = 0.003, 95% CI = [19.00, 89.75]; marginal effect %-change, time window 40–60 min: 54.39, *SE* = 18.28, *p* = 0.003, 95% CI = [18.56, 90.21]). A control analysis on ADR power differences between infusion groups in a subset of patients that could be tested within 3 days after the bleed showed comparable results (see supplemental table III for marginal effects and supplemental figure VIII for model residuals).

Further analyses also investigated relative changes from baseline. There was a relative increase in ADR from baseline in the sodium nitrite arm for the first and third time window (predictive margin %-change, time window 0–20 min: 31.77, *SE* = 12.22, *p* = 0.009, 95% CI = [7.83, 55.72]; predictive margin %-change, time window 40–60 min: 35.80, *SE* = 13.00, *p* = 0.006, 95% CI = [10.33, 61.28]).

The saline arm showed a relative decrease in ADR from baseline in the second time window (predictive margin %-change, time window 20–40 min = − 33.19, *SE* = 12.18, *p* = 0.006, 95% CI = [− 57.05, − 9.32]).

In line with the results for the ADR, there was a difference in alpha power change between the sodium nitrite and the saline group for all time windows (Fig. 2b). There was more alpha power change with sodium nitrite infusion (marginal effect %-change, time window 0–20 min: 37.03, *SE* = 12.21, *p* = 0.002, 95% CI = [13.10, 60.95]; marginal effect %-change, time window 20–40 min: 46.51, *SE* = 12.39, *p* < 0.001, 95% CI = [22.23, 70.78]; marginal effect %-change, time window 40–60 min: 28.76, *SE* = 12.57, *p* = 0.022, 95% CI = [4.12, 53.41]).

The sodium nitrite arm showed a relative increase in alpha power from baseline for all time windows (predictive margin %-change, time window 0–20 min: 26.64, *SE* = 8.32, *p* = 0.001, 95% CI = [10.33, 42.95]; predictive margin %-change, time window 20–40 min: 23.93, *SE* = 8.66, *p* = 0.006, 95% CI = [6.96, 40.90]; predictive margin %-change, time window 40–60 min: 20.69, *SE* = 8.95, *p* = 0.021, 95% CI = [3.15, 38.24]).

There was a relative decrease from baseline in alpha power with saline infusion in the second time window (predictive margin %-change, time window 20–40 min: - 22.58, *SE* = 8.28, *p* = 0.006, 95% CI = [− 38.81, − 6.35]).

There was a difference in changes in delta power between infusion groups during the second time window (Fig. 2c), where delta power change was larger within the saline group (marginal effect %-change, time window 20–40 min: 28.50, *SE* = 13.70, *p* = 0.037, 95% CI = [1.65, 55.35]).

In addition, the saline arm showed a relative increase in delta power from baseline for the second and the third time window (predictive margin %-change, time window 20–40 min: 35.82, *SE* = 10.00, *p* < 0.001, 95% CI = [17.98, 53.65]; predictive margin %-change, time window 40–60 min: 19.81, *SE* = 10.00, *p* = 0.029, 95% CI = [1.97, 37.64]).

Sedation was associated with marginally lower ADR (difference %-change: − 52.75, *SE* = 29.37, *p* = 0.073, 95% CI = [− 110.31, 4.82]) and significantly lower alpha (difference %-change: − 49.51, *SE* = 18.76, *p* = 0.008, 95% CI = [− 86.28, − 12.74]) power changes. No effect of baseline was observed on any EEG measure.

### Individual EEG Data in Patients Who Developed DCI

**Fig. 3:**
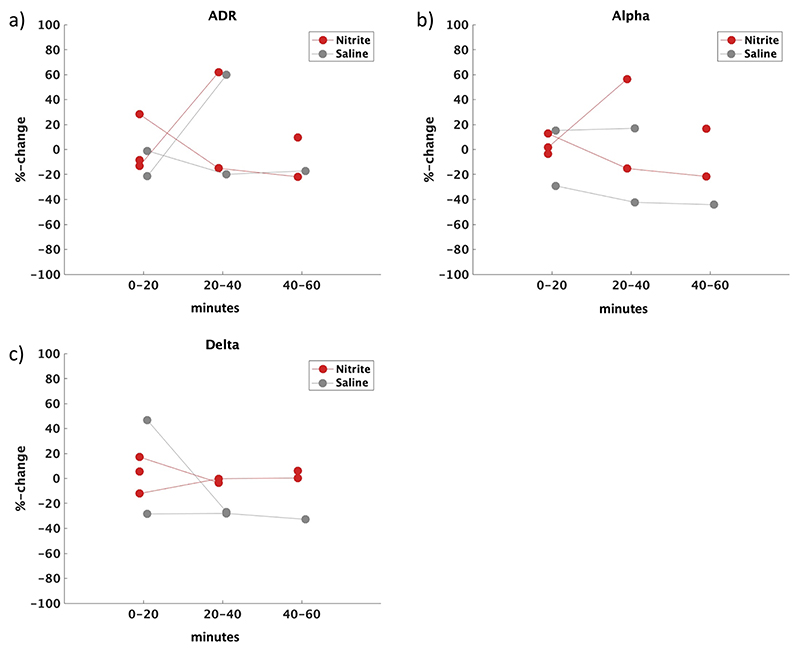
Individual EEG data in patients who developed DCI. Percentage change ADR (a), alpha (b) and delta (c) power from baseline during nitrite (red) and saline (grey) infusion for individual time windows

Data from the five patients who developed DCI are presented on an individual basis. There was no obvious difference between infusion groups for either time window with ADR, alpha or delta power changes (Fig. 3a–c).

## Discussion

Our key finding in our main study group (patients that did not develop DCI) was that intravenous sodium nitrite led to an increase in ADR and alpha power above baseline and saline placebo. We suggest that these changes reflect the action of NO and relate to a generally less ischaemic brain pattern. Our findings confirm and extend animal studies showing the importance of the NO pathway after SAH and during ischaemic conditions. They provide a basis for future studies investigating NO signalling after human SAH.

Previous research has connected decreases in alpha and increases in delta power to ischaemic conditions [30]. In the context of DCI after SAH, similar relationships have been shown [29], where increases or decreases in alpha and ADR were even predictive of a less or more ischaemic brain pattern [31, 32]. Higher alpha power and lower delta power during rest have also both explicitly been linked to increased cerebral perfusion and oxygen uptake [25–28, 30, 39]. Therefore, we suggest that increases in alpha and ADR power with the present investigation could relate to nitrite-mediated reductions of ischaemia and potentially increases in cerebral perfusion.

Case studies suggest that alpha and delta frequencies can follow drug action aimed at improving cerebral ischaemia after SAH [29, 40] and thrombolysis after ischaemic stroke [41]. Infused sodium nitrite is well suited to alter brain states after cerebral damage. It can safely be administered [23] and is converted to NO in hypoxic and acidic conditions [16, 17], which can be observed after SAH [6, 42]. It can support cerebral autoregulation after SAH [43] and, crucially, helps preserve and maintain cerebral microcirculation and protect nerve cells after cerebral insult [11, 44]. Whereas clinical studies on the effect of NO availability as measured by NO metabolites after SAH are still inconclusive [12–15], animal studies using experimental models suggest beneficial effects of nitrite administration on cerebral angiographic vasospasm after SAH [22] and neuroprotective effects with ischaemic conditions [20, 21]. However, NO metabolites mostly sampled from the cerebrospinal fluid represent an indirect and regionally unspecific measure. They may not be a good proxy for actual NO availability in injured brain areas. Instead of concentrating on indirect measures of global NO distribution, we probed the neural effects of NO donor administration in SAH patients by using EEG markers relating to immediate neural processing, cerebral ischaemia and regional cerebral blood flow.

We extend and clarify the findings of Garry et al. [24] who investigated a small cohort of critically ill patients with SAH in an intensive care environment using an identical sodium nitrite infusion. The main findings were robust within-subject increases in the ADR from baseline in those patients who did not develop DCI, but a decrease in ADR in those who subsequently developed DCI. However, Garry et al.’s study was uncontrolled, and all patients were sedated, intubated and mechanically ventilated, and thus sicker than the present cohort.

We observed an increase in ADR for nitrite versus saline infusion across the entire sample of patients. As we expected a more robust effect of nitrite infusion in patients who do not develop DCI (and the clear majority of patients do not develop DCI [6]), our study was designed and powered for this patient group. Individual datasets of patients who developed DCI showed variable responses with no obvious relation to infusion type. For patients who did not develop DCI, we replicated a within-subject ADR increase with sodium nitrite infusion relative to baseline. Most importantly, we also observed a between-subject difference in ADR changes with sodium nitrite versus saline infusion, where sodium nitrite infusion was related to relative ADR increases. Therefore, we clarified that EEG changes were not merely an effect of time. We conducted a randomised, double-blind mechanism study. Therefore, we are confident that we did not introduce an experimenter or patient-related bias of treatment or expectation. Patients featured generally rather good-grade WFNS scores. They were nevertheless representative of the subarachnoid haemorrhage stroke population in Oxfordshire, as we did not restrict patients to a specific grade of stroke severity. Importantly, it has been shown that even good-grade patients can experience secondary deterioration [45] and functional deficits which relate to less favourable outcome [46]. More than 30% of good-grade patients do not return to work after years [47].

We further clarified nitrite action by investigating frequency specific contributions to ADR power changes. Crucially, we observed a within-subject increase in alpha power from baseline during sodium nitrite infusion and a between-subject difference in alpha changes, where sodium nitrite infusion was related to a relative increase in alpha. Hence, ADR power changes reflect alpha power changes.

Previous research has found decreases in alpha power with fentanyl, propofol and midazolam administration [36, 38]. In our study, alpha power changes from baseline were decreased when patients were sedated. Therefore, the present study shows the importance of controlling for sedation in patient studies using EEG, even if relative EEG power is investigated.

Delta power showed an increase from baseline with saline infusion and was temporarily higher with saline compared to nitrite infusion. This was mirrored by a pattern of mean and temporarily significant decreases in alpha and ADR power with saline infusion. As absolute baseline power between the two infusion groups was not obviously different, respectively accounted for in our model, and patients were otherwise resting, we speculate that the changes in alpha and delta activity from baseline during saline infusion could relate to declining vigilance. The first infusion time window already included time points from at least 20–40 min of recording after EEG setup. A decrease in alpha activity and more prominent delta activity have been related to lower levels of vigilance [48, 49].

Unlike alpha power, delta power was comparably less sensitive to nitrite infusion with the present investigation, and ADR power changes did not show superior responsivity to alpha power changes. Stroke severity in our patient population was lower compared to patients in other investigations where a prominent role of delta or ADR in ischaemia was suggested. There, patients were stuporous or comatose [29] or featured higher WFNS scores [24, 32]. Machado et al. [50] put forward for consideration that delta power in ischaemic stroke patients would rather relate to an ischaemic core region, while alpha power would reflect oedema, tissue at risk and the penumbra. Accordingly, delta power changes have been related to more prominent reductions in cerebral blood flow compared to alpha power changes [30]. It could thus be argued that patients in our study featured comparably more residually intact tissue, which could be reflected in a lack of delta reactivity. Alternatively, nitrite might generally only act in less damaged and residually functioning tissue. Future studies comparing alpha and delta responses to nitrite infusion in higher versus lower grade SAH patients will need to clarify the possibility of an interaction between nitrite action and SAH lesion severity.

Importantly, Franko et al. [33] did not find a change in alpha, delta or ADR power with sodium nitrite versus saline infusion in healthy participants suggesting that the EEG changes observed with the present investigation are disease related. As already discussed, nitrite is reduced to NO specifically well in hypoxic and acidic environments [16, 17]. Therefore, infused nitrite could preferentially be converted to NO in the injured brain, e.g. after SAH. In injured but residually functional tissue, NO could best exert its neuroprotective effects, including an increase in regional cerebral perfusion, which is reflected in EEG. In contrast, an increased level of nitrite in normoxic tissue of healthy brains would not have a large effect on brain perfusion and the EEG would not change accordingly.

Despite the fact that NO has been proposed to promote various beneficial effects on damaged tissue, including an improvement of cerebral blood flow at the microcirculatory [11, 44] level and with larger vessels [43], and a reduction in platelet aggregation, inflammation and Ca^2+^-mediated cytotoxicity [16], our experimental methods do not allow for an explanation of the effects of nitrite infusion beyond the level of a reduction in ischaemic processes and an increase in cerebral perfusion [25–30]. Subsequent investigations may use denser electrode setups, potentially in combination with other techniques [51], to further characterise the site of nitrite action.

While none of our patients was diagnosed to suffer from DCI at or before the time of our study, we conducted the study within a maximum of 5 days after the bleed. The time frame reflects our effort to recruit as many patients as possible in order to obtain a representative sample of subarachnoid haemorrhage patients. We believe that the results from our group analyses nevertheless reflect EBI disease mechanisms, as patients were on average tested within 3 days after the ictus. A focussed control analysis in a subset of patients who could be tested within 3 days after the bleed showed comparable results.

To complement this research, it will be necessary to study the effect of nitrite infusion in a larger group of SAH patients who develop DCI. In particular, it will be mechanistically informative to determine if EEG markers of ischaemia and perfusion can also be modulated in this population of SAH survivors and if a different dosing scheme of sodium nitrite infusion will be necessary.

Other studies aiming toward the use of sodium nitrite as a therapeutic have infused sodium nitrite in healthy participants for 2 days [52] or SAH patients for 2 weeks [23]. Although sodium nitrite was infused at comparably lower doses in these investigations, the long-term infusion dosing scheme in SAH patients [23] suggested that blood nitrite levels were at ceiling with the highest dose administered. We cannot exclude an effect of short-term sodium nitrite infusion on outcome with the present research as our study was not powered for investigating patients who develop DCI. However, we deliberately administered sodium nitrite only comparably very briefly and for 1 h, as we did not aim to treat patients but merely probe the cerebral response to an NO donor after SAH. Crucially, we could indeed show a beneficial change in electrophysiological markers of ischaemia in patients who did not develop DCI. Future clinical trials will need to investigate the diagnostic [24] and therapeutic potential of short- and long-term sodium nitrite infusion.

Our findings may help render such studies safer. In addition to beneficial and neuroprotective effects of NO after cerebral insult, detrimental and cytotoxic effects relating to secondary brain injury are also known, and these depend on timing and regional availability of NO [9, 53]. Whereas increased NO at the vascular bed of residually functioning tissue after SAH could be neuroprotective, excessive NO in critically injured brain tissue may further cell damage. Therefore, clinical investigations using NO donors after SAH should tightly monitor NO action on neuronal functioning. EEG is safe, non-invasive, cheap and not dependent on active patient involvement. Most importantly, EEG directly reflects neuronal processing and is related to ischaemia and cerebral perfusion. Therefore, EEG might help to determine optimal levels of nitrite infusion to maximise patient safety [23]. Our research shows that the EEG can be used to study effects of short-term NO donor infusion after SAH and provides a mechanistic basis for future EEG-aided clinical trials employing NO donors.

## Conclusions

To conclude, we studied the effects of short-term sodium nitrite infusion during EBI after SAH using EEG. Sodium nitrite infusion compared to saline control infusion resulted in clear relative increases in the ADR and alpha power in patients who did not develop DCI. We suggest that these EEG changes reflect NO action and relate to a change toward a less ischaemic brain pattern. In future studies, using EEG to determine the effect of NO donors after SAH should improve mechanistic understanding of the NO pathway and could further the development of novel diagnostics and therapeutics.

## Supplementary Material

Supplementary material

## Figures and Tables

**Table 1:** Characteristics of patients without DCI grouped by infusion type

	Nitrite (*n* = 14)	Saline (*n* = 14)
Age, years, mean (range)	53 (31–69)	47 (29–61)
Female, *n* (%)	9 (64)	9 (64)
Time from bleed, hours, *mean* (*SD*)	64 (23)	68 (23)
Fisher scale, *n* (%)		
1	1 (7)	1 (7)
2	3 (21)	3 (21)
3	6 (43)	3 (21)
4	4 (29)	7 (50)
WFNS score on admission, *n* (%)		
1	10 (71)	10 (71)
2	2 (14)	4 (29)
4	1 (7)	0 (0)
5	1 (7)	0 (0)
GCS on study day, *n* (%)		
E1VTM1	3 (21)	0 (0)
15	11 (79)	14 (100)
Sedation, *n* (%)	3 (21)	0 (0)
ADR at baseline, *mean* (*SD*)	0.28 (0.24)	0.35 (0.25)
Alpha at baseline, *mean* (*SD*)	4.07 (2.55)	5.84 (3.69)
Delta at baseline, *mean* (*SD*)	25.34 (24.94)	20.29 (12.82)

**Table 2:** Individual characteristics of patients who developed DCI

	Patient 1	Patient 2	Patient 3	Patient 4	Patient 5
Nitrite or saline, N/S	N	S	N	S	N
Age, years	51	66	43	53	63
Female or male, F/M	M	M	F	F	F
Time from bleed, hours	60	45	90	96	82
Fisher scale	2	4	4	2	2
WFNS score on admission	4	2	1	2	1
GCS on study day	14	15	15	15	15
Sedation, 0/1	0	1	0	0	0
ADR at baseline	0.27	0.05	0.44	0.23	0.2
Alpha at baseline	3.67	3.76	6.14	8.33	4.91
Delta at baseline	13.71	72.97	13.97	36.39	25.14

## Data Availability

The data that support the findings of this study are available from the corresponding author upon reasonable request.

## Code Availability

Data processing scripts for this study are available from the corresponding author upon reasonable request.
