# Supplementary material for: Beneficial Effect of Sodium Nitrite on EEG Ischaemic Markers in Patients with Subarachnoid Haemorrhage
Source: Transl Stroke Res. Author manuscript; Available in PMC 2022 Apr 1. (PMC8918451; doi:10.1007/s12975-021-00939-9)
Supplement: Supplementary material [file EMS140017-supplement-Supplementary_material.docx]

**Supplemental materials for “Beneficial effect of sodium nitrite on EEG ischaemic markers in patients with subarachnoid haemorrhage”**

**Supplemental methods**

**Assessments**

WFNS score and Fisher scale were assessed after admission. On the day of the intervention, demographics, medical history, GCS, EEG recordings and vital functions were collected. Clinical progress was monitored from the day of the intervention until discharge to detect drug-related adverse events, where neurological function was specifically monitored on the day of the intervention and at discharge. In a small subset of patients Mini Mental State Examination (MMSE) and Oxford Cognitive Screen (OCS) scores were collected. Likewise, transcranial Doppler recordings could be acquired in a subset of patients.

**EEG**

The EEG was acquired continuously with the Porti 7 system (Twente Medical Systems International, Oldenzaal, The Netherlands) using OpenViBE acquisition software according to the International 10-20 system with a 512-Hz sampling rate. Brain potentials were recorded from 12 electrodes: Fp1, Fp2, F3, Fz, F4, T3, Cz, T4, P3, Pz, P4, Oz. In addition, left and right mastoid recordings were taken. Patients were bed-bound and their vital functions were monitored. For several patients, access to the back of the head was impaired for practical or medical reasons. In few cases, electrode O1 was used instead of electrode Oz. The ground electrode was placed on the forehead.

EEG data was processed in Matlab version 2016 (The MathWorks) using custom written functions and the FieldTrip toolbox [1] version 20170720. Individual raw data sets were screened for data points where no signal was recorded. Electrodes were then referenced to the average left and right mastoid recording. The data was demeaned, bandpass filtered (0.1-40 Hz) and epoched into segments of 2 seconds [2]. The absolute difference between the highest and lowest potential recorded within an epoch was determined. Excessively noisy epochs containing an absolute potential difference of more than 70 µV and epochs where data points where missing as determined by raw data screening were excluded from further analysis. The signal was decomposed using Hanning tapers (fixed time window: 1s, range: 0.5-1.5 s, step size: 1/512*50 s). Frequency power was estimated in steps of 0.5 Hz and averaged within the alpha (8-12 Hz) and delta (1-3.5 Hz) and frequency bands [2]. For each epoch and frequency band, power was averaged across time points and electrodes obtaining a single alpha and delta power measure per epoch. The ratio of alpha to delta power (ADR) was calculated. The alpha, delta and ADR signals were then averaged across epochs separately within a 20-minute baseline time window before and within a 60-minute or three successive 20-minute infusion time windows after the onset of the infusion, as predetermined in a statistical analysis plan (<https://osf.io/ywa9r/>).

**Supplemental tables and figures**

**Supplemental table I: Number of datasets per electrode**

The number of datasets per electrodes was biased toward frontal and medial electrodes, as for several patients access to the back of the head was challenging for practical and medical reasons (e.g. due to positioning of ventricular drains or extreme headache). A reduced set of electrodes was attached to these patients. In few cases, electrode O1 was used instead of electrode Oz. Crucially, electrode availability was largely comparable between infusion groups of patients who did not develop DCI.

**Supplemental table II: Number of data sets per time window**


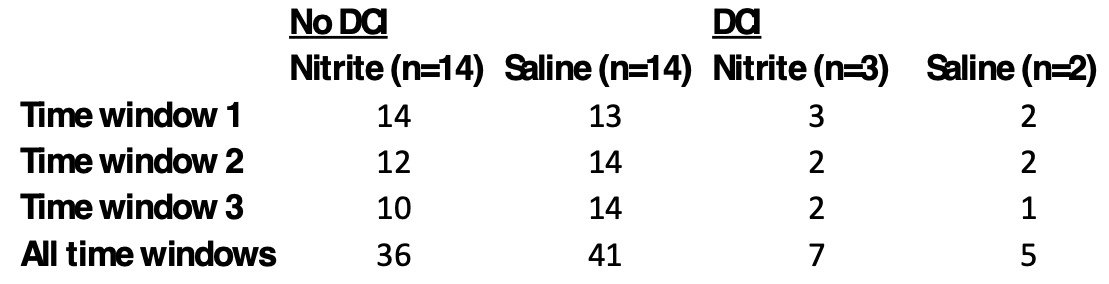


There was a minimum of 30 epochs per time window amounting to one minute of data. This is in accordance with the amount of data used per time point in Garry et al.’s study [3]. If a time window of an individual patient did not feature at least 30 epochs, this time window was excluded for that patient.

**Supplemental figures I-III: Simple linear regression across all patients**

**Supplemental figure I:** Model residuals for simple regression across all patients, ADR: residuals (a) and residual quantiles vs. inverse normal quantiles (b).

**Supplemental figure II:** Model residuals for simple regression across all patients, alpha: residuals (a) and residual quantiles vs. inverse normal quantiles (b).

**Supplemental figure III:** Model residuals for simple regression across all patients, delta: residuals (a) and residual quantiles vs. inverse normal quantiles (b).

**Supplemental figures IV-VI: Simple linear regression in patients who did not develop DCI**

**Supplemental figure IV:** Model residuals for simple regression in patients who did not develop DCI, ADR: residuals (a) and residual quantiles vs. inverse normal quantiles (b).

**Supplemental figure V:** Model residuals for simple regression in patients who did not develop DCI, alpha: residuals (a) and residual quantiles vs. inverse normal quantiles (b).

**Supplemental figure VI:** Model residuals for simple regression in patients who did not develop DCI, delta: residuals (a) and residual quantiles vs. inverse normal quantiles (b).

**Supplemental figures VII-XII, table III-V: Multilevel linear regression in patients who did not develop DCI**

**Supplemental figure VII:** Model residuals for multilevel regression in patients who did not develop DCI, ADR: residuals (a) and residual quantiles vs. inverse normal quantiles (b).

**Supplemental figure VIII:** Model residuals for multilevel regression in patients who did not develop DCI and were tested within 3 days of the bleed, ADR: residuals (a) and residual quantiles vs. inverse normal quantiles (b).

**Supplemental table III: ADR power changes for multilevel regression in patients who did not develop DCI and were tested within 3 days of the bleed: Marginal effects**

**Supplemental figure IX:** Model residuals for multilevel regression in patients who did not develop DCI, alpha: residuals (a) and residual quantiles vs. inverse normal quantiles (b).

**Supplemental figure X:** Model residuals for multilevel regression in patients who did not develop DCI, delta: residuals (a) and residual quantiles vs. inverse normal quantiles (b).

The diagnostic plots show one extreme observation for both alpha and delta power change. As diagnostic plots for ADR power change do not show extreme observations and we aimed to have a consistent data set for all three models, the paper presents model results for alpha and delta power including all observations. Removing extreme observations does not have a major effect on model results. Related predictive margins and marginal effects are presented in supplemental tables IV (alpha power changes) and V (delta power changes). Related model diagnostics are presented in supplemental figures XI (alpha power changes) and XII (delta power changes).

**Supplemental table IV: Alpha power changes for multilevel regression in patients who did not develop DCI, extreme observation removed: Predictive margins and marginal effects**


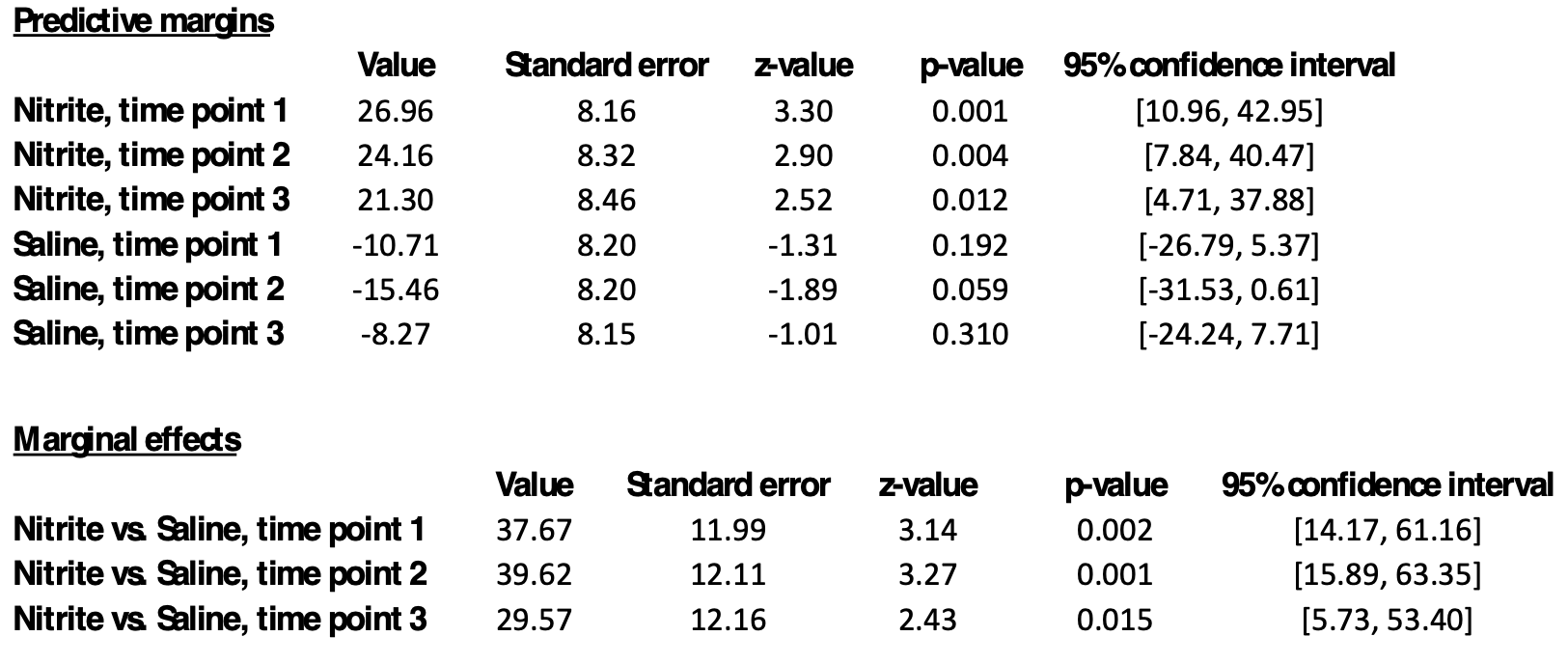


**Supplemental table V: Delta power changes for multilevel regression in patients who did not develop DCI, extreme observation removed: Predictive margins and marginal effects**


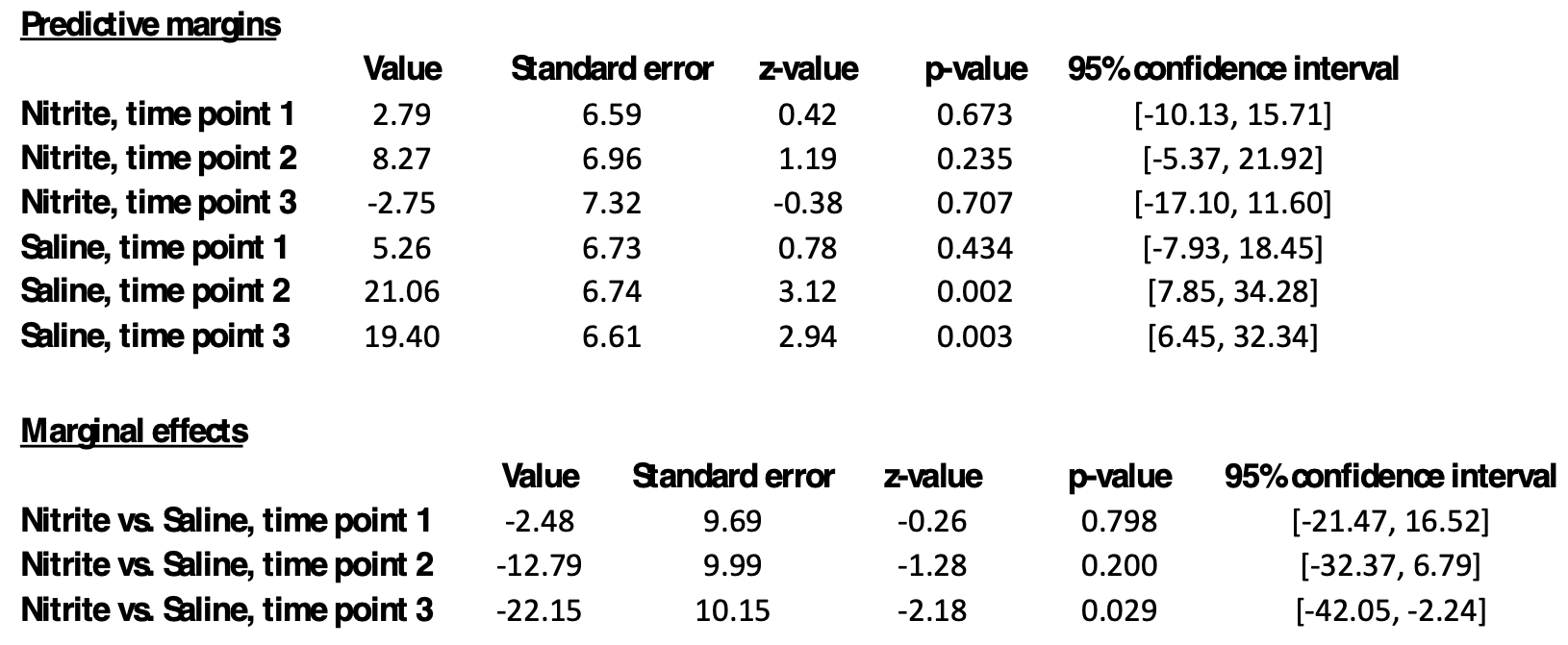


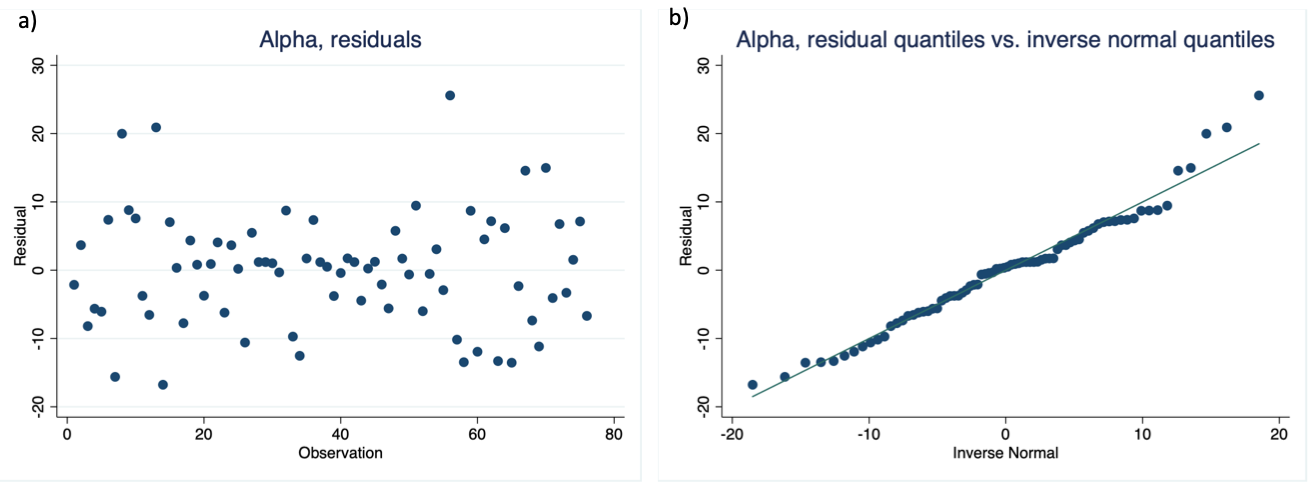


**Supplemental figure XI:** Model residuals for multilevel regression in patients who did not develop DCI, alpha, extreme observation removed: residuals (a) and residual quantiles vs. inverse normal quantiles (b).


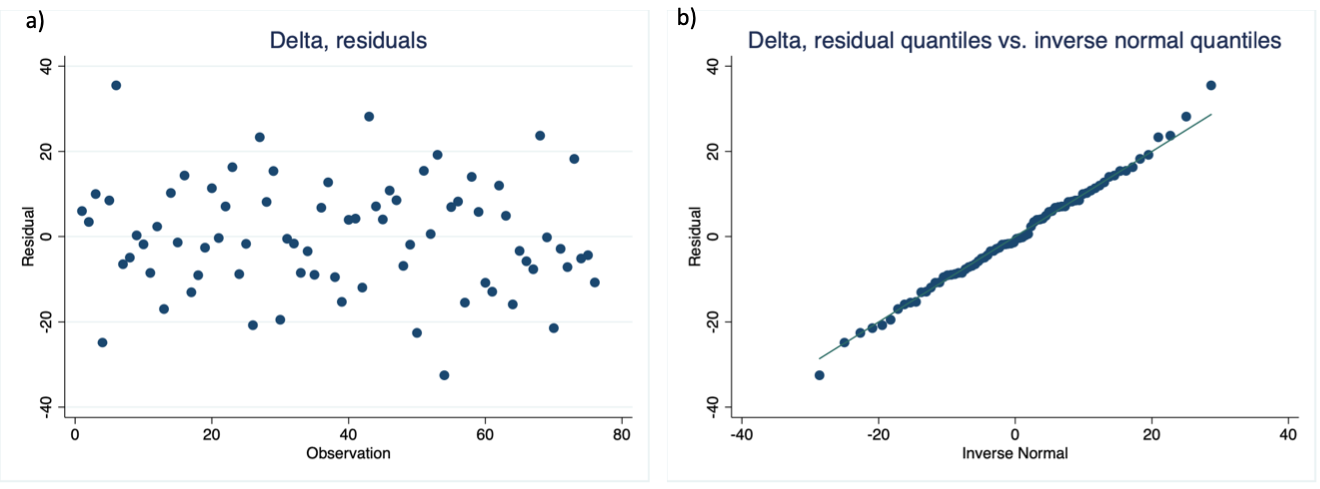


**Supplemental figure XII:** Model residuals for multilevel regression in patients who did not develop DCI, delta, extreme observation removed: residuals (a) and residual quantiles vs. inverse normal quantiles (b).

**References:**

1. Oostenveld R, Fries P, Maris E, Schoffelen J-M. FieldTrip: Open source software for advanced analysis of MEG, EEG, and invasive electrophysiological data. Comput Intell Neurosci. 2011;2011.

2. Franko E, Ezra M, Crockett DC, Joly O, Pattinson K. Effect of nitrite on the electroencephalographic activity in the healthy brain. Nitric Oxide - Biol Chem [Internet]. Elsevier; 2019;90:47–54. Available from: https://doi.org/10.1016/j.niox.2019.06.002

3. Garry PS, Rowland MJ, Ezra M, Herigstad M, Hayen A, Sleigh JW, et al. Electroencephalographic Response to Sodium Nitrite May Predict Delayed Cerebral Ischemia After Severe Subarachnoid Hemorrhage. Crit Care Med [Internet]. 2016;44:e1067–73. Available from: http://insights.ovid.com/crossref?an=00003246-201611000-00032
